# Endocannabinoid System as Therapeutic Target of PTSD: A Systematic Review

**DOI:** 10.3390/life11030214

**Published:** 2021-03-09

**Authors:** Luca Steardo, Elvira Anna Carbone, Giulia Menculini, Patrizia Moretti, Luca Steardo, Alfonso Tortorella

**Affiliations:** 1 Psychiatric Unit, Department of Health Sciences, University Magna Graecia of Catanzaro, Viale Tommaso Campanella, 115, 88100 Catanzaro, Italy; 2 Department of Medical and Surgical Sciences, University Magna Graecia of Catanzaro, Viale Tommaso Campanella, 115, 88100 Catanzaro, Italy; elvira.carbone@libero.it; 3 Department of Psychiatry, University of Perugia, Piazzale Lucio Severi, 1, 06132 Perugia, Italy; giuliamenculini@gmail.com (G.M.); patrizia.moretti@unipg.it (P.M.); alfonso.tortorella@unipg.it (A.T.); 4 Department of Physiology and Pharmacology, Faculty of Pharmacy and Medicine, Sapienza University of Rome, Piazzale Aldo Moro, 5, 00185 Rome, Italy; lucasteardo@uniroma1.it; 5 Department of Psychiatry, Giustino Fortunato University, 12, 82100 Benevento, Italy

**Keywords:** endocannabinoids, CBD, cannabidiol, THC, Δ^9^-tetrahydrocannabinol, nabilone, PTSD, PTSD treatment, systematic review

## Abstract

Post-Traumatic Stress Disorder (PTSD) is a complex disorder involving dysregulation of stress-related hormones and neurotransmitter systems. Research focused on the endocannabinoid system (eCBS) for anxiety and stress regulation, cognitive and emotional responses modulation and aversive memories extinction, leading to the hypothesis that it could represent a possible alternative treatment target for PTSD. In this systematic review, we summarize evidence about the efficacy and safety of medicinal cannabidiol (CBD), Δ^9^-tetrahydrocannabinol (Δ^9^-THC), and nabilone in PTSD treatment. The PRISMA statement guidelines were followed. A systematic literature search was conducted in MEDLINE/PubMed, Scopus and Web of Science by two independent researchers, who also performed data extraction and quality assessment. Among the initial 495 papers, 234 were screened for eligibility and 10 were included. Studies suggested that different medicinal cannabinoids at distinct doses and formulations could represent promising treatment strategies for the improvement of overall PTSD symptomatology as well as specific symptom domains (e.g., sleep disorders, arousal disturbances, suicidal thoughts), also influencing quality of life, pain and social impact. Although there is a robust rationale for treatment with drugs that target the eCBS and the results are promising, further studies are needed to investigate the safety and efficacy profile of their prolonged use.

## 1. Introduction

Post-Traumatic Stress Disorder (PTSD) is a psychiatric disorder that may occur in people after experiencing or witnessing a traumatic event. This nosographic entity was previously included in the Diagnostic and Statistical Manual of Mental Disorders, fourth edition (DSM-IV) as an anxiety disorder, whereas in the latest edition (DSM-5) PTSD was classified under a category named “Trauma and stress-related disorders” [1,2]. With a prevalence varying up to 5% in high-income countries, PTSD is often associated with significant comorbidity rates, relevant risk of chronicization, and substantial economic burden [3].

In the context of a maladaptive response to a traumatic stressor [4,5], individuals can develop symptoms such as persistent intrusive thoughts associated with the traumatic event, incessant avoidance of stimuli related to the traumatic event, negative changes in cognition and mood linked to the event, and significant and long-lasting alterations in arousal and reactivity attributable to the traumatic event. These symptoms can last for more than one month or persist for several months, causing significant distress due to poor life quality, reduced social skills, and functioning problems [1].

Although PTSD is caused by a psychologically traumatic environmental event and consequently it is considered a psychological phenomenon, many studies have indicated biological abnormalities, not observed in controls, which have been regarded either as risk factors for the development of the disease or as potential targets for therapeutic interventions. There have been detected changes across different stress hormones and neurotransmitter systems in the pathophysiology of PTSD. Serotoninergic, noradrenergic, glutamatergic, GABAergic, neuro-peptidergic systems and dysfunctions of the hypothalamus-adrenal axis have been reported to contribute to the disease onset and progression, even if no alteration of a single system taken individually can explain by itself the complex pathobiology underlying the disorder. In this context, and mainly based on findings from preclinical studies, a growing interest in terms of neurochemical interactions has been attracted by the endocannabinoid system (eCBS), since it makes up an extensive interconnected network of neuromodulators that controls synaptic neurotransmitter release. As it has been involved in several physiological and pathophysiological processes including synaptic plasticity, pain, memory processes, stress, and emotion regulation, its potential implication in the pathophysiology of PTSD has been regarded of considerable interest [6]. Indeed, the eCBS plays an important role in regulating anxiety and stress and is involved in the modulation of cognitive and emotional responses [7], as well as in the extinction of aversive memories [8]. This system is essential for the development of synaptic plasticity in response to endogenous and environmental salient events [9,10], determining specific learning and emotional responses related to traumatic experiences [11]. Endocannabinoids regulate affective and emotional states and participate in memory consolidation, retrieval, and extinction [12,13]. It has been demonstrated that the learning processes regarding aversive memories are dysregulated after a traumatic event, as occurs in subjects affected by PTSD [14]. Evidence suggests that the hypothalamic–pituitary–adrenal (HPA) system, which is essential for stress adaption, also plays a role in the pathophysiology of the disorder. Indeed, the HPA axis is chronically activated in patients with PTSD [15]. There is a bidirectional and functional interplay between eCBS and HPA systems: the eCBS can modulate the HPA axis function restraining the effects of severe stress on its activation [16,17,18], whilst the release of glucocorticoids in response to a stressor contributes to eCBS signaling deficiencies, thus facilitating PTSD symptoms [19]. 

Given the heterogeneity of the disorder and its economic and social burden, PTSD represents a challenge for safe and effective treatment [20]. Despite more psychological treatment approaches for PTSD [21,22], the Food and Drug Administration (FDA) approved only two pharmacological treatments for PTSD, the Selective Serotonin Reuptake Inhibitors (SSRIs) paroxetine and sertraline [23] that do not display certain effectiveness in producing a clinical remission and may be responsible for several side effects [24,25], which can sometimes reduce treatment compliance [26]. 

Recent research has focused on the eCBS as a possible alternative target to treat PTSD [27,28]. Evidence highlighted a reduction of endocannabinoid levels [29] and cannabinoid receptor type 1 (CB1) receptors up-regulation in the brain of PTSD patients [30]. Thus, the use of components derived from *Cannabis sativa* as cannabidiol (CBD) and Δ^9^-tetrahydrocannabinol (Δ^9^-THC) has drawn increasing interest as a possible alternative approach to treat PTSD. To reinforce this assumption several studies support cannabis use as self-medication to cope with PTSD symptoms [4,31]. 

While CBD is known to bind the G-protein-coupled receptor GPR55, the transient receptor potential of vanilloid type-1 channel (TRPV1), the 5-HT1a receptor, and the α3 and α1 adrenergic receptors, we will focus on the CB1 and CB2 receptors, since they are relevant to the Central Nervous System (CNS) [32]. Preclinical and clinical studies suggested that CBD, acting also as a negative allosteric modulator of endogenous ligands of CB1 and CB2, may present a beneficial effect in PTSD treatment, enhancing the consolidation of explicit fear extinction and attenuating aversive memories of the traumatic event [33,34,35,36]. Moreover, Δ^9^-THC, a CB1 and CB2 partial agonist, showed a safe and well-tolerated profile in chronic PTSD with a significant improvement in global symptom severity, sleep quality, frequency of nightmares, and hyperarousal symptoms [37]. More recently, nabilone, a synthetic cannabinoid that activates CB1, was reported to reduce the frequency and intensity of nightmares in PTSD patients [38,39,40,41].

Despite promising preliminary evidence, no reviews to our best knowledge have systematically summarized the effectiveness of cannabinoids or cannabino-mimetic in the treatment of PTSD. Previous literature reviewed the evidence about the eCBS as a potential target for PTSD treatment and prevention, but without following a systematic approach and focusing also on pathophysiological correlates of the disorder [28,42,43]. In consideration of what is stated above, the present systematic review is aimed at summarizing the existent evidence on medicinal cannabinoids (e.g., THC, CBD, nabilone) in the treatment of PTSD in humans, critically analyzing and discussing both the efficacy and safety of these treatment approaches.

## 2. Materials and Methods

The present review was conducted following the Preferred Reporting Items for Systematic Reviews and Meta-Analyses (PRISMA) statement [44].

### 2.1. Literature Search

A systematic search of the electronic databases MEDLINE/PubMed, Scopus, and Web of Science was performed from inception to 23 November 2020 by entering the following search string: (((((((cannabidiol) OR cannabinoid) OR cannabinol) OR endocannabinoid) OR tetrahydrocannabinol) OR nabilone) OR palmitoyl-ethanolamide) AND ((post-traumatic stress disorder) OR PTSD) AND treatment.

Two investigators (EAC and GM) independently conducted the literature search, title/abstract screening, and full-text review. The reference list of selected articles was hand-screened to search for additional literature. Discrepancies were resolved through consensus and, whenever a final decision could not be achieved, a third investigator was consulted (LS jr).

### 2.2. Study Selection

Original studies investigating the effectiveness of medicinal cannabinoids in the treatment of PTSD diagnosed according to the DSM criteria were deemed eligible for inclusion, according to the PICO methodology. Literature considering specific symptoms of PTSD as treatment outcomes was also evaluated for the present review, with no language limits. Grey literature was included whenever sufficient information was provided. Articles presenting only an opinion or hypothesis without empirical investigation, reviews, letters to the editor, and commentaries were excluded. Case series were included if sufficient information was given. Studies reporting data on genetic and molecular aspects in animal models were not deemed eligible for the present review. As for studies with overlapping samples, more than one article was considered for inclusion if reporting on different aspects of the considered outcomes. 

### 2.3. Quality Assessment 

Considering the heterogeneity of included studies, the Grading of Recommendations, Assessment, Development and Evaluation (GRADE) approach was used to rate the quality of the evidence [45]. This was done by two reviewers (EAC and GM) and disagreements were resolved via discussion with one further reviewer (LS jr). The quality of evidence was rated as “high”, “moderate,” “low,” or “very low” based on GRADE rating standards. A “high-quality” rating indicates that future research is very unlikely to change existing evidence and that the true effect is similar to the estimated effect; a “moderate-quality” and a “low-quality” rating indicates that future research may change/is likely to change the evaluation results, respectively; a “very low-quality” rating indicates that it is highly uncertain about the existing evidence and that the true effect is likely to be substantially different from the estimated effect.

### 2.4. Data Extraction

Two blind researchers (EAC and GM) performed data extraction. In order to address the objectives of the review, the following information was extracted from the included papers: first author name, year and country of publication, analyzed period, study design, study sample (number, age, and gender), treatment provided, comparator group (e.g., placebo, active), study measure and outcomes. Reviewers extracted data independently from each relevant study and data was checked by all reviewers in case of discrepancies. 

### 2.5. Risk of Bias Assessment

The quality of the evidence provided by the eligible studies was assessed by the two independent researchers (GM and EAC). The risk of bias for randomized controlled trials (RCTs) was evaluated with the Revised Cochrane Risk of Bias Tool (RoB 2.0) [46]. 

## 3. Results

### 3.1. Search Results

**Figure 1:**
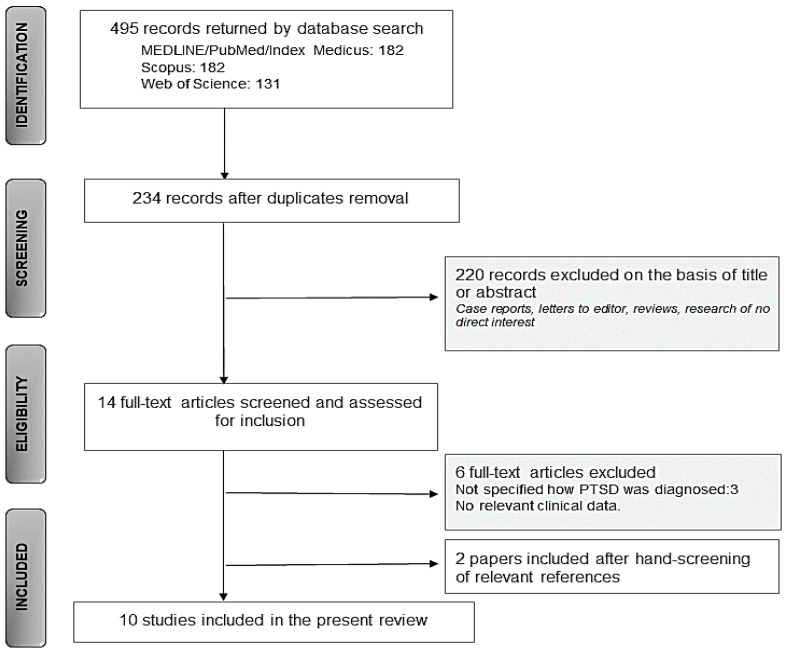
The PRISMA flow chart.

The initial search returned 495 records (MEDLINE/PubMed = 182, Scopus = 182, Web of Science = 131). Among these, 261 were identified as duplicates and were subsequently excluded. Title and abstract screening were performed for the remaining 234 papers, 14 of which were included in the further evaluation. The full-text examination led to the selection of 8 papers. The hand-screening of references led to the inclusion of two additional records. Subsequently, 10 papers were deemed eligible for the present review (see flowchart in Figure 1). 

### 3.2. Content Results

All studies were published over a period from 2009 to 2019 and mainly conducted in the US or Canada. Diagnosis of PTSD was made using DSM-IV or DSM-5 criteria. We included four retrospective studies [40,47,48,49], two observational studies [50,51], two open-label studies [37,38], and two randomized, double-blind, placebo-controlled studies [39,52]. Most of the studies included male subjects and two studies evaluated veterans or military personnel [39,48]. The mean age of participants ranged between 32 and 52 years [37,40,52]. Treatment with CBD only [47], THC only [37,52], medical cannabis (THC and CBD at variable percentages) [48,49,50,51], and nabilone [38,39,40] was evaluated in included studies considering different formulations (e.g., liquid oil spray, capsule), gradual titration and mean dose. The mean dose of CBD was 9 mg/d (range 1–16) for liquid oil spray formulation and 25 mg/d (range 25–100) for capsule [47]; THC was used at 7.5 mg in capsule formulation or oil 0.1 cc bid raising to 0.2 cc bid (0.1 cc = 2.5 mg) [37] while medical cannabis (THC and CBD at variable percentages) was started at 1 g/d and titrated based on clinical response to a maximum dose of 10 g/d [48,49] and a mean dose of 1.2–3 g/d [50,51]. The start dose of nabilone was 0.5–2 mg and titrated weekly to a maximum of 3–4 mg/d (range 0.5–6 mg) [38,39,40]. Only two studies used a placebo comparator group [39,52]. The treatment had variable duration with a minimum of three weeks [37] and a maximum of 43 weeks [40] of drug administration in the acute phase [52]. Regarding psychiatric measures, changes in PTSD Checklist for the DSM-5 (PCL-5) [47], Post-Traumatic Checklist–Civilian Version (PCL-C) [40], Clinician-Administered PTSD Scale (CAPS) [37,39,49,51], Clinical Global Impressions (CGI) [37,39], Pittsburgh Sleep Quality Index (PSQI), Nightmare Effects Survey (NES), Nightmare Effects Survey (NFQ) [37], Well Being Questionnaire (WBQ) [39], and Quality of Life Scale (QOLS) [51] scores were evident. Only one study evaluated the modulation of the amygdala and pre-frontal activation under an acute low dose of THC in patients with and without PTSD and healthy controls (HC) [52]. Considering outcomes, a reduction in PTSD symptoms (e.g., nightmares, intrusive thoughts, anxiety, arousal) as measured by psychometric scores was detected, as well as a decrease of suicidal thoughts. A decrease of PTSD social impact score from 6.6 to 2.7 as reported in clinical charts [48] and an improvement in the quality of life in QOLS score [51] and WBQ score [39] after treatment was also demonstrated. Therefore, results showed that cannabinoids (e.g., THC, CBD, nabilone) may be effective in treating PTSD symptoms as clinical response and psychometric scores demonstrated. Furthermore, acute administration of a low dose of cannabinoid acted on corticolimbic responses to threat-related processing. Data from included studies are reported in Table 1.

### 3.3. Quality and Risk of Bias Assessment

According to the GRADE approach, only two studies presented high quality, whilst most of the included research showed low-to-moderate quality. Detailed information concerning quality assessment is included in Table 1. According to the RoB 2.0 tool, there were some concerns about the possible risk of bias for included RCTs, mainly affecting the randomization process and data reporting (Table 2). Indeed, in the study with randomized, double-blind, placebo-controlled cross-over design [39] information about the sequence allocation was not available, whilst in the randomized, double-blind, placebo-controlled, between-subjects study [52] outcome data was not available for all randomized subjects.

## 4. Discussion

Studies included in the present review suggested that different medicinal cannabinoids at distinct doses and formulations could represent promising treatment strategies for PTSD-related symptoms, also presenting an impact on the quality of life in this population. Particularly, not only did treatment with cannabinoids determine an overall improvement of PTSD symptomatology [37,39,40,47,48,49,51], but it also influenced specific symptom domains, namely sleep disorders [37,38,39], arousal disturbances [37], and suicidal thoughts [48]. Noteworthy, a significant reduction of pain [48,51] and social impact [48], as well as an improvement in quality of life and well-being [39,51], were demonstrated.

A better understanding of the possible mechanisms through which such treatments could influence the clinical expression of PTSD is of particular interest since most medications used in clinical practice have the main aim of relieving symptom severity without specifically targeting underlying biological pathways [53]. 

To note, CB receptors are differently expressed in brain areas and nervous cells: CB1 receptors are highly expressed in the cortex, basal ganglia, hippocampus, hypothalamus, and cerebellum while CB2 receptors are mostly expressed in the immune and gastrointestinal cells and they may modulate central and peripheral regions [9]. CBD acts on both CB1 and CB2 receptors with lower affinity for Δ^9^-THC, producing less adverse effects (e.g., tachycardia, sedation, hunger) but significantly decreasing fear-memory consolidation and anxiety symptoms of PTSD, possibly due to its action on limbic and paralimbic areas [54,55]. CBD can also exert an agonistic action on the 5-HT1a receptor that may explain its antiemetic and anxiolytic-like properties, on the GPR55 enhancing neuronal excitability, and on the TRPV1, highly localized in the hippocampus, reducing anxiety and aversive memories [32]. The inverse agonist action on CB2 may also explain the anti-inflammatory effects [56]. Δ^9^-THC acts through neuronal presynaptic CB1 receptors as a partial agonist and inhibits directly or indirectly ongoing neurotransmitter release such as dopamine, glutamate, and acetylcholine [55] in the nucleus accumbens, prefrontal cortex, hippocampus, and amygdala with a consequential reduction in hypervigilance, anxiety, insomnia, nightmares, and extinction deficits related to PTSD [57,58]. Nabilone, a synthetic THC analog, is a CB1 receptor agonist and has been demonstrated to reduce recurrent nightmares, flashbacks, and improve sleep time even if the exact mechanism is not yet understood [38,39,40,41,59]. 

The efficacy of medicinal cannabinoids in reducing overall PTSD symptomatology might indeed find a possible explanation in their mechanism of action, given their modulatory influence on different neurotransmitter systems, as demonstrated by the presence of CB receptors in pre-synaptic GABAergic and glutamatergic terminations, but also in noradrenergic, serotoninergic, and dopaminergic axon terminals [27,60]. 

Noteworthy, PTSD pathophysiology relies on abnormalities that cut across different neural circuits, involving distinct neurotransmitters at the same time [61]. Given the heterogeneity of PTSD symptomatology, belonging to several domains as stated in the diagnostic criteria, molecules that could target more than one system could represent adequate candidates for acting on different clinical features [28], thus reducing overall illness severity. 

Besides, the eCBS plays a key role in the process of extinguishing aversive memories, which is mainly mediated by CB1 and could be thus facilitated by CBD administration, as already suggested by animal models, with further confirmation in human studies [34,62]. Similar mechanisms could also explain the improvement in arousal symptom clusters, that appear to be activated by emotional processing of fear-elicited stimuli and altered responses to trauma-related cues [63]. Interestingly, only one among the included studies considered the administration of CBD at variable doses [47], whilst in the remaining studies subjects were treated with different formulations of medicinal cannabis containing THC and CBD at different percentages, or synthetic compounds. Consequently, aversive memories extinction is not expected to be the only mechanism possibly explaining the efficacy of medicinal cannabis in reducing PTSD symptoms. The effect on global PTSD symptomatology might be mediated by the reduction of HPA axis hyperactivation following eCBS signaling enhancement. This could be related to the expression of CB receptors in brain areas that modulate stress, as well as emotional responses to fear and reward, such as the amygdala, pre-frontal cortex, and hippocampus [64]. Particularly, CB1 in the pre-frontal cortex plays a significant role in threat-related responses by modulating the amygdala region [65]. Noteworthy, one of the selected studies showed how the administration of medicinal THC in PTSD subjects could influence cortico-limbic circuitry, also reducing amygdala reactivity in response to potentially threatening stimuli [52]. This result should hopefully be replicated for CBD and synthetic *Cannabis*-derived compounds, to evaluate whether slightly different actions on eCBS receptors may underpin different effects on brain circuitry. Moreover, future studies should better clarify functional correlates of treatment with medications acting on the eCBS to provide further explanations about their mechanism of action in reducing PTSD symptoms. Furthermore, specific symptom clusters were targeted by treatment with medicinal cannabis in some of the included studies. Particularly, sleep problems such as PTSD-related nightmares were significantly improved by THC oil and nabilone at a maximum dose of 3–6 mg. This result may be of clinical relevance since nightmares are not typically targeted by medications that appear to be efficient in treating other PTSD symptoms [66]. The efficacy of *Cannabis* derivates in ameliorating sleep disorders was already elucidated by animal studies, with specific effects on sleep duration and depth varying based on means and site of administration (e.g., intraperitoneal, amygdala, brain ventriculus, etc.) [67,68]. Diverse effects of CBD were over time detected depending on medication doses, with higher doses up to 160 mg/day increasing sedative-hypnotic effects [69,70]. Such effects were hypothesized to be mediated by the monoaminergic system since CBD appeared to increase the expression of c-Fos in the dorsal raphe nuclei [71]. Furthermore, CBD can increase Anandamide (AEA) concentration by blocking the AEA membrane transporter (AMT) or the Fatty Acid Amide Hydrolase (FAAH) enzyme, which catalyzes AEA hydrolysis, increasing time of sleep and of slow-wave phase that is physiologically fostered by this endogenous CB1 ligand [72]. Nevertheless, most preclinical research focused mainly on the administration of CBD, which acts as a negative allosteric modulator of CB1 [73], while the included studies considered effects of TCH and its derivates, possibly operating as a partial agonist of the receptor [74]. In consideration of their different actions on endogenous receptors, further studies on PTSD populations should better clarify different actions of THC and CBD on sleep disorders. This could be of particular interest also due to the frequent association that specific arousal disturbances may present with nightmares and sleep abnormalities, with evidence for nabilone improving such disturbances in non-PTSD populations [75].

Notably, pain indexes were significantly reduced after treatment with nabilone at a maximum dose of 6 mg/d and medicinal cannabis with 20–25% THC component at 2–3 g/d. The use of medicinal cannabis for chronic pain symptoms finds wide support in the medical literature given the modulatory effects of the eCBS on inflammation and pain-processing pathways in the central and peripheric nervous systems, mainly relying on CB2 expression in peripheral tissues and immune cells [76,77,78,79]. Pain is frequently associated with PTSD clinical features, with evidence about mutual influences that such symptoms may exert. Future research focused on this specific comorbidity may elucidate whether treatment with cannabinoids could represent a favorable treatment strategy for selected PTSD subjects with specific comorbidities.

Despite promising results on the improvement of quality of life, as well as reduction of the social impact of PTSD, the use of medicinal cannabinoids in this population could also present negative consequences on general well-being due to their abuse and dependence potential [59,80]. Indeed, subjects affected by PTSD demonstrated the tendency to use cannabis as a form of self-medication due to its anxiolytic and sedative properties [57,81,82], also displaying high rates of comorbid substance-related disorders [83]. Except for cannabidiol which is a phyto-derivative of cannabis devoid of any psychotropic effect, the recreational use of cannabis, particularly synthetic compounds with high THC content, may elicit the emergence of psychotic and anxiety symptoms, as well as cognitive dysfunctions [84,85,86]. Conversely, side effects of medicinal cannabinoids (e.g., dizziness, headache, fatigue, gastrointestinal problems) presented mild-to-moderate severity and were relatively well-tolerated, supporting the assumption that subjects with PTSD may consider them less burdensome than approved medications [87]. Future research should hopefully clarify the long-term effects of such treatments to provide further data concerning their safety profile.

Evidence reported by the included studies presents several limitations. First, most of the considered research is based on small sample sizes, with restricted generalizability of findings. Moreover, heterogeneous samples determine major limitations in the among-study comparability of results. Included studies show low-to-moderate quality and moderate-to-high risk of bias in most cases, which suggests that further studies are expected to address methodological issues to provide solid bases for the use of medicinal cannabinoids in clinical practice. In addition, the retrospective or observational design of most of the included studies precludes conclusions about causality. Only a few RCTs were conducted and comparators were used only in two cases, despite repeated calls for controlled studies that may elucidate the effectiveness of new medications in PTSD treatment [61,88]. Finally, medications were assumed for short periods in most cases and no follow-up data were thus provided. Other randomized, placebo-controlled studies designed or head-to-head comparator studies are therefore needed.

## 5. Conclusions

Post-Traumatic Stress Disorder is a particularly heterogeneous clinical entity since it does not invariably exhibit in all subjects the same variety of symptoms and neither do they have similar severity. Moreover, it frequently occurs in comorbidity with other physical or mental disorders. Although there is a robust rationale for the treatment with drugs active, directly or indirectly, on the cannabinoid system and the results to date are promising, further studies are needed to investigate the safety and efficacy profile of their prolonged use. Hopefully, continued research could provide clinicians with novel therapeutic options for a disease that currently is treated with drugs of limited efficacy.

## Figures and Tables

**Table 1:** The main characteristics of included studies.

Author	Year and Country	Period	Study Design	Study Sample	Treatment	Comparator Group	Measures	Outcome
Elms et al. [47]	2019, US (Colorado)	8 weeks	Retrospective study Grade: *	N = 11 (8 F, 3 M); mean age 39.91 ± 17.39; DSM-5 diagnosis of PTSD	N = 1: 9 mg/d (mean dose, range 1–16) CBD liquid oil spray; N = 4: 25 mg/d (mean dose, range 25–100) CBD capsule; N = 6: both formulations	None	Changes in PCL-5 score	Decline in PCL-5 mean scores of 21% (from 51.82 to 40.73) in 91% subjects at 4 weeks. Further decrease of 9% (to 37.14) in 75% subjects at 8 weeks.
Rabinak et al. [52]	2019, US (Michigan)	Acute study	Randomized, double-blind, placebo-controlled, between-subjects study Grade: ****	N = 71 (36 F, 35 M) age 20–45; N = 22: DSM-5 diagnosis of PTSD; N = 24: TEC; N = 25: HC	THC 7.5 mg capsule	Placebo	Threat-processing paradigm at fMRI (amygdala, mPFC/rACC activation and functional connectivity)	In the PTSD group THC lowered threat-related bilateral amygdala activity (drug x group interaction left: F(1,65): 5.131, *p* = 0.027; right: F(1,65) = 4.456, *p* = 0.039), increased mPFC activation (drug x group interaction F(2,65) = 4.887, *p* = 0.011), increased mPFC-right amygdala functional coupling (F(1,65) = 8.181, *p* = 0.006).
Smith et al. [48]	2017, Canada	2 years, variable treatment duration and time to follow-up	Retrospective study Grade: ***	N = 100 veterans (3 F, 97 M); mean age 43; DSM-5 diagnosis of PTSD	Medical cannabis (THC and CBD at variable percentages), start dose 1 g/d, self-titrated based on clinical response, maximum dose 10 g/d	None	Changes in PTSD-related symptoms, pain, and social impact, scored 1–10 as reported in clinical charts	Decrease of PTSD aggregate symptoms score from 7 to 2.9 (59% reduction, ES 1.5, *p* < 0.0001), decrease of suicidal thoughts score from 4.1 to 0.9 (77% reduction, ES 1, *p* < 0.0001), decrease of pain score from 6.6 to 3.4 (48% reduction, ES 1.5), decrease of PTSD social impact score from 6.6 to 2.7 (59% reduction, ES 1.2, *p* < 0.0001) after treatment.
Cameron et al. [40]	2014, Canada	43 months	Retrospective study Grade: **	N = 104 males; mean age 32.7 (range 19–55); 91% of which affected by DSM-IV-TR PTSD	Nabilone, mean initial dose 1.4 mg/d (range 0.5–2), mean final dose 4 mg/d (range 0.5–6), mean length of treatment 11.2 weeks (range 1 day–36 weeks)	None	Changes in PCL-C scores	Significant reduction of PLC-C scores (54.7 ± 13 vs 38.8 ± 7.1, *p* = 0.001) after treatment, consistent with a reduction from moderate to mild-borderline symptoms.
Greer et al. [49]	2014, US (California)	30 months	Retrospective study Grade: **	N = 80 adults; DSM-IV diagnosis of PTSD	Medical cannabis (THC and CBD at variable percentages), start dose 1 g/d, self-titrated based on clinical response, maximum dose 10 g/d	None	Changes in CAPS scores	Decrease in total CAPS score (22.5 ± 16.9 vs 98.8 ± 17.6, *p* < 0.0001) in subjects using cannabis. Significant reductions in CAPS symptom cluster scores (Cannabis × Cluster: F(2,158) = 39.87, *p* < 0.0001).
Jetly et al. [39]	2014, Canada	Two periods of 7 weeks separated by a period of 2 weeks	Randomized, double-blind, placebo-controlled crossover study Grade: ****	N = 10 military male personnel; age 18–65; DSM-IV-TR diagnosis of PTSD, current distressing nightmares, and difficulty falling/staying asleep as evaluated by CAPS	Nabilone 0.5 mg (start dose) weekly titrated to a maximum of 3 mg	Placebo	Changes in CAPS Recurrent Distressing Dreams and Difficulty Falling or Staying Asleep items, CGI-C, PTSD Dream Rating Scale, WBQ scores, Sleep Diary	Significant reduction of CAPS Recurring and Distressing Dream Frequency (−1.9 ± 1.3 vs −0.4 ± 1.4, *p* = 0.05) and Intensity (−1.7 ± 1.3 vs −0.6 ± 1.1, *p* = 0.06) scores, significant lower CGI-C (1.9 ± 1.1 vs 3.2 ± 1.2, *p* = 0.05) score, significant increase of WBQ (49.3 ± 21.6 vs 23 ± 17.2, *p* = 0.04) score after treatment with nabilone.
Roitman et al. [37]	2014, Israel	3 weeks	Non-randomized, open-label, adjusted doses, study Grade: ***	N = 10 (3 F, 7 M); mean age 52.3 ± 8.3; DSM-IV PTSD diagnosis	TCH oil 0.1 cc (=2.5 mg) bid, after 2 days raised to 0.2 cc (=5 mg) bid	None	Changes in CAPS, CGI, PSQI, NFQ, NES scores	Significant decrease in CAPS arousal (32.3 ± 4.73 vs 24.3 ± 9.11, *p* < 0.02), CGI-S (6 ± 0.47 vs 4.9 ± 0.99, *p* < 0.02), NFQ frequency of nightmares (0.81 ± 0.55 vs 0.44 ± 0.41, *p* < 0.04), NES (32.2 ± 11.29 vs 22.9 ± 8.7, *p* < 0.002), PSQI (17.20 ± 2.65 vs 13.9 ± 4.48, *p* < 0.05) scores after treatment with THC.
Bonn-Miller et al. [50]	2013, US (California)	Not specified	Cross-sectional study Grade: **	N = 217 (26.7% F, 73.3% M); mean age 41.2 ± 14.9 (range 18–74 years); 40 (18.9%) affected by PTSD	Medical cannabis, flexible-dose (mean use: 3 times/d, 9–12 g/week)	None	Subjective help received from medical cannabis as measured by a 5-point-likert scale	Traumatic intrusions predicted cannabis helpfulness (beta 0.22, *p* < 0.01), as well as the use of cannabis for social anxiety problems (*p* < 0.004).
Reznik [51]	2012, Israel	3 years	Naturalistic observational study Grade: **	N = 167 25 pure PTSD, 43 PTSD + clinical depression, 88 PTSD + chronic pain	Medicinal cannabis (20–25% THC), range 2–3 g/d	None	Changes in CAPS, QOLS, CGI-I scores	Significant improvement in QOLS and pain scores in most cases, with some positive changes in CAPS scores. The majority of improved subjects belonged to comorbidity groups.
Fraser [38]	2009, Canada	2 years of clinical observation; flexible duration of treatment with nabilone (depending on the clinical response)	Non-randomized, open-label study Grade: ***	N = 47 (27 F, 20 M); mean age 44 ± 9; DSM-IV-TR diagnosis of PTSD, treatment-resistant nightmares	Nabilone 0.5 mg (start dose) before bedtime, then titrated; doses were kept below 6 mg/d	None	Changes in the intensity of PTSD-related nightmares	34 (72%) subjects experienced total cessation or significant reduction of nightmares. Nabilone discontinuation was successful in 4 (8%) subjects, whilst the others experienced a recurrence of nightmares.

**Note:** CAPS: Clinician-Administered PTSD Scale; CBD: Cannabidiol; CGI: Clinical Global Impressions; CGI-C: Clinical Global Impressions—Change; CGI-I: Clinical Global Impressions—Improvement; CGI-S: Clinical Global Impressions—Severity; DSM-IV: Diagnostic and Statistical Manual of Mental Disorders, 4th Edition; DSM-IV-TR: Diagnostic and Statistical Manual of Mental Disorders, 4th Edition, Text Revision; DSM-5: Diagnostic and Statistical Manual of Mental Disorders, 5th Edition; ES: Effect Size; fMRI: Functional Magnetic Resonance Imaging; HC: Healthy Control; mPFC: Medial Pre-Frontal Cortex; NES: Nightmare Effects Survey; NFQ: Nightmare Frequency Questionnaire; PCL-C: Post-Traumatic Checklist—Civilian Version; PCL-5: PTSD Checklist for the DSM-5; PSQI: Pittsburgh Sleep Quality Index; PTSD: Post-Traumatic Stress Disorder; QOLS: Quality of Life Scale; rACC: Rostral Adjacent Cingulate Cortex; TCH: delta-9-tetrahydrocannabinol; TEC: Trauma-Exposed-Control; WBQ: Well Being Questionnaire. GRADE: * very low; ** low; *** moderate; **** high.

**Table 2:** Evaluation of the risk of bias for Randomized Studies using the RoB 2.0 Tool.

References	Overall Risk ^a^	Randomization	Intervention	Missing Data	Outcome Measurement	Reported Results
Rabinak et al. (2019) [52]	+/−	−	−	+/−	−	−
Jetly et al. (2014) [39]	+/−	+/−	−	−	−	−

^a^ Risk of bias: low (−), some concerns (+/−), high (+).
